# Gait Biomechanical Differences in the Anterior Cruciate Ligament Reconstructed and Contralateral Limb: A Systematic Review with Meta-Analysis

**DOI:** 10.3390/healthcare13243304

**Published:** 2025-12-16

**Authors:** Heidar Sajedi, Elif Aydın, Mehmet Şirin Güler, Selahattin Akpınar, Ali Esmaeili, AmirAli Jafarnezhadgero, Kate E. Webster

**Affiliations:** 1 Department of Exercise and Sports Science for Disabled People, Faculty of Health Science, International Science and Technology University, 01-982 Warsaw, Poland; 2 Physical Education and Sports Department, Sport Science Faculty, Ardahan University, 75000 Ardahan, Türkiye; elifaydin@ardahan.edu.tr; 3 Department of Physical Education and Sport Teaching, Faculty of Sports Sciences, Sarıkamış Campus, Kafkas University, 36500 Kars, Türkiye; mehmet.guler@kafkas.edu.tr; 4 Faculty of Sport Sciences, Düzce University, 81620 Düzce, Türkiye; selahattinakpinar@duzce.edu.tr; 5 Department of Sport Biomechanics, Faculty of Educational Science and Psychology, University of Mohaghegh Ardabili, Ardabil 56199-11367, Iran; alismaeelisky76@gmail.com (A.E.); amiralijafarnezhad@gmail.com (A.J.); 6 School of Allied Health, Human Services and Sport, La Trobe University, Bundoora, VIC 3086, Australia; k.webster@latrobe.edu.au

**Keywords:** walking, kinetics, kinematics, ACL surgery

## Abstract

Background: In this systematic review with meta-analysis, we aimed to compare the kinematic and kinetic variables of the involved limb with the contralateral limb in individuals who had undergone an anterior cruciate ligament reconstruction (ACLR) recorded during walking from short-term (<6 months) to mid-term (6–<12 months) and long-term (≥12 months) periods after surgery. Methods: Five electronic databases (Scopus, PubMed, EMBASE, PEDro, CENTRAL) were systematically searched for articles potentially eligible for inclusion from inception until November 2025. Biomechanical gait patterns were assessed short-term (<6 months), mid-term (6–<12 months), and long-term (≥12 months) post-surgery. Gait biomechanics were extracted from the included articles. Comparisons were made between the affected limb and the contralateral limb. Standardized mean differences (SMDs) with 95% confidence intervals (CI) were computed using a random-effects model. Results: The systematic search revealed 3522 hits, and according to a priori defined in-/exclusion criteria, 32 studies with male and female individuals aged 18–55 years involving 1026 participants were included. Meta-analysis indicated that the peak knee flexion angle was significantly lower in the ACLR compared to the contralateral limb (19 studies: small SMDs = −0.39, 95% CI −0.58 to −0.19, *p* < 0.0001, I^2^ = 66%). More specifically, the peak knee flexion angle was 2.63° (95% CI −3.81 to −1.44) lower in the ACLR compared to the contralateral limb. The analysis of time post-surgery revealed significant differences in the short-term (four studies: large SMDs = −1.14, 95% CI −1.61 to −0.67, *p* < 0.00001, I^2^ = 56%) and mid-term (five studies: small SMDs = −0.52, 95% CI −0.74 to −0.29, *p* < 0.0001, I^2^ = 0%) periods after surgery but not for the long-term follow-up (10 studies: small SMDs = −0.10, 95% CI −0.27 to 0.07, *p* = 0.26, I^2^ = 32%). Meta-analysis indicated that the peak knee flexion moment was significantly lower in the ACLR compared to the contralateral limb (11 studies: small SMDs = −0.37, 95% CI −0.59 to −0.14, *p* = 0.0001, I^2^ = 46%). A lower peak knee flexion moment was observed in the ACLR limb for both less than 12 months (three studies: moderate SMDs = −0.76, 95% CI −1.44 to −0.07, *p* = 0.03, I^2^ = 66%) and over 12 months (eight studies: small SMDs = −0.25, 95% CI −0.43 to −0.07, *p* = 0.01, I^2^ = 46%) after surgery time points compared to the contralateral limb. Conclusion: These findings suggest a time-dependent compensatory mechanism, where protective adaptations (e.g., reduced flexion/extension moments) may initially offload the reconstructed limb, with some asymmetries resolving over time. Clinically, these results underscore the need for rehabilitation strategies tailored to address phase-specific deficits, promoting symmetrical loading and functional recovery.

## 1. Introduction

After anterior cruciate ligament (ACL) rupture, ACL reconstruction (ACLR) is a common surgical treatment to restore joint stability and help patients return to their pre-injury mobility levels. ACLR restores joint stability [1,2] but does not attenuate the risk of knee osteoarthritis [3,4]. In fact, 30 to 80% of patients develop osteoarthritis within 10–15 years post-ACLR [3,4,5], and other joint injuries are evident as early as 1–2 years post-ACLR [6,7]. Moreover, it has been shown that ACLR results in altered walking patterns as indicated by knee loading asymmetries [8,9,10].

Decreased knee flexion range of motion and extensor moments during the loading phase of walking [8,9,10,11,12] and limb asymmetries in knee extensor moments [13] have been reported by several studies in patients who have undergone ACLR. Such alterations have been attributed to the progression of knee joint osteoarthritis in this already vulnerable population [14,15].

There are, nonetheless, several conflicting results with regard to lower limb joint kinematics and joint moments post-ACLR that have been reported in cross-sectional studies [16,17,18,19,20,21,22,23,24,25,26]. For instance, while Ferber et al. [19] reported greater hip flexion angle in ACLR patients, Noehren et al. [22] have shown lower peak hip flexion angle in ACLR populations compared to the healthy control group during walking. Also, Webster et al. [24] reported lower peak vertical ground reaction force in ACLR patients, while Noehren et al. [22] have shown greater peak vertical ground reaction force in ACLR populations compared to the healthy control group. The underlying reasons for the discrepancy in findings may reflect between-study variability in patient population characteristics (e.g., graft type, gender, and time post-ACLR), comparator (healthy controls vs. contralateral knees), data acquisition and processing, variables of interest (e.g., mean or peak values, range of motion), and data presentation (e.g., normalization). Thus, it seems timely to aggregate the available findings in the literature by taking potential moderators into account.

Previously, researchers have conducted systematic reviews with meta-analysis on the impact of ACLR on patients’ gait patterns [27,28,29,30,31]. However, these studies were limited in that Hart et al. [30] and Shi et al. [27] conducted meta-analyses only on knee sagittal and transverse planes kinematics and moments during level walking. Further, Hart et al. [28] and Gokeler et al. [29] qualitatively reported knee kinematics and moments without conducting any meta-analyses. These reviews reported the presence of gait alterations in the sagittal, frontal, and transverse planes after ACLR [28,29]. In 2017, Slater et al. [31] conducted a meta-analysis in order to compare the three-dimensional lower extremity kinematics and kinetics of walking among individuals with ACL deficiency, individuals with ACLR, and healthy control participants from 3 to 64 months after ACLR. However, Slater et al. [31] did not evaluate the ACLR limb and contralateral limb biomechanical differences. A clearer understanding of the alternation of three-dimensional lower-extremity biomechanics from the time point post-ACLR through short-term (<6 months), mid-term (6–<12 months), and long-term (≥12 months) periods may help clinicians and researchers better target evaluation and intervention to improve short-, mid-, and long-term outcomes after ACLR. Therefore, it is important to identify lower limb kinematics and kinetics during level walking after ACLR to understand its potential implications for post-ACLR rehabilitation. This systematic review and meta-analysis aimed to compare the kinematic (e.g., joint angles) and kinetic (e.g., joint moments and ground reaction forces) variables for each joint of the ACLR-affected limb and the contralateral (healthy) limb from short-term to mid- and long-term periods after surgery during walking.

## 2. Materials and Methods

The standard PRISMA (Preferred Reporting Items for Systematic Reviews and Meta-Analyses) guidelines were followed when conducting this systematic review with meta-analysis [32]. The protocol for this systematic review with meta-analysis was registered with PROSPERO on 19 March 2025. In the present systematic review with meta-analysis, biomechanical gait patterns were assessed at <6 months, 6–<12 months, and ≥12 months post-surgery.

### 2.1. Eligibility Criteria

EndNote 20 software (Bld 14672, Clarivate, Philadelphia, PA, USA) was used for systematic search and the processing of potentially eligible papers. A PECOS (participants, exposure, comparators, outcomes, and study design) approach was applied to define inclusion and exclusion criteria (Table 1) a priori [33]. To be eligible for inclusion in this meta-analysis, articles had to be published in peer-reviewed journals in the English language. Prospective cohort studies were excluded only when they lacked isolated cross-sectional biomechanical data comparing ACLR and contralateral limbs. Cohorts providing baseline or single-time-point gait data were eligible and included. Articles not written in the English language were excluded (Table 1).

### 2.2. Systematic Search

A comprehensive literature search was conducted in five electronic databases: PubMed, Scopus, Web of Science, PEDro, and the Cochrane Central Register of Controlled Trials (CENTRAL) from inception to 25 November 2025. The search syntax was created using the PECOS scheme and free-text keywords, as well as medical subject headings (MeSH terms). Keywords and MeSH terms were combined using a Boolean search syntax and the operators AND, OR. The complete Boolean search strategies for each database, including all free-text terms, MeSH terms, field tags, truncation symbols, and applied filters, are provided in Supplementary Materials File S1: Tables S1–S5 to ensure full reproducibility. The PubMed strategy was developed first using the PECOS framework and was subsequently adapted for each database.

No language, publication status, or study design filters were applied at the database-query stage. The following date-of-last-search timestamps were recorded: PubMed (13 November 2025), Scopus (25 November 2025), Web of Science (14 November 2025), PEDro (15 November 2025), and CENTRAL (15 November 2025).

Gray-literature screening was performed in Google Scholar, ScienceDirect, ClinicalTrials.gov, and ProQuest Dissertations. For Google Scholar, the first 200 results sorted by relevance were screened, consistent with recommendations for gray-literature reproducibility. For ClinicalTrials.gov, we searched all registered trials involving ACL reconstruction and gait assessment without date restrictions. Conference proceedings and dissertation abstracts were included if full-text biomechanical data were available. Reference lists of all included studies were also manually screened.

### 2.3. Study Selection

Titles and abstracts were reviewed by two authors (A.E. and A.J.) to identify potentially eligible studies according to the a priori defined inclusion and exclusion criteria. When titles and abstracts did not provide sufficient information, full texts were examined.

### 2.4. Quality Assessment

The methodological quality of the studies included was evaluated by the same two authors (A.E., A.J.) using a modified version of the Downs and Black checklist for non-randomized controlled trials [34]. The modified checklist includes 18 questions with eight reporting items (items 1, 2, 3, 4, 5, 6, 7, 10), two items for external validity (items 11 and 12), four items for internal validity (Bias) (items 15, 16, 18, 20), three items for internal validity-confounding (items 21, 22, 25), and one item for power (item 27) (Supplementary Materials File S2: Tables S6 and S7). The items were scored as 0 (“no” and “unable to determine”) and 1 (“yes”), except for item 5 for the principal confounders, which was scored 0 (“no”), 1 (“partially”), and 2 (“yes”). The overall quality score of each study was calculated based on a percentage of the maximum score (19). In cases where there were discrepancies in the authors’ rating of the quality scores, consensus was reached through discussion. Studies with quality scores of 75% or higher were considered high quality, those between 60% and 74% were classified as moderate quality, and those with 60% or lower were categorized as low quality [35]. Studies were not excluded from meta-analysis based on quality. Inter-rater agreement for the quality assessment was evaluated using Cohen’s kappa (κ) statistic for each domain of the modified Downs and Black checklist. The level of agreement was quantified as slight (0.00 to 0.20), fair (0.21 to 0.40), moderate (0.41 to 0.60), substantial (0.61 to 0.80), and almost perfect (0.81 to 1.00) [35]. Disagreements were resolved with a predefined adjudication rule; a third senior reviewer (H.S.) adjudicated disagreements that persisted after discussion. Quality scores were used for descriptive purposes and were not applied as analytic weights. The most frequently observed limitations involved incomplete confounder control (e.g., walking-speed prescription, graft type) and inadequate reporting of gait-acquisition protocols.

### 2.5. Data Collection

Two reviewers (A.E. and A.J.) independently extracted all relevant data (participants, control, number of participants, gender, graft, time since surgery, time since injury, walking protocol, and outcomes related to kinematic and kinetic data) from the included articles. For studies requiring plot digitization, two independent digitizations were performed and averaged. Inter-extractor reliability was excellent (ICC = 0.94). Peak angles and joint excursion values were extracted from group averages and reported as kinematic variables. Joint moments and ground reaction force were reported as kinetic variables. In studies where numerical values were not reported, we attempted to obtain them directly through the corresponding author or a freeware web-based plot digitizer [36] to obtain data from graphs. To extract data from figures, a freeware web-based plot digitizer was used (https://plotdigitizer.com/webplotdigitizer-alternative (accessed on 16 May 2025)). Several included studies reported more than one postoperative time point for the same cohort. To avoid violating the assumption of independence, we extracted and analyzed only one time point per study for each meta-analysis. This ensured that each pooled effect included only independent comparisons. When a study reported multiple postoperative assessments, we selected the time point that corresponded to the predefined windows (<6 months, 6–<12 months, ≥12 months) and matched the most common reporting pattern across studies. This approach avoids inflating sample size and weighting and maintains methodological consistency across the review. Next, we categorized the data based on the term after surgery (short term (<6 months) to mid-term (6–<12 months), and long term (≥12 months)) and compared the kinematic and kinetic variables for each joint for both the ACLR limb and contralateral limb. To enhance transparency regarding outcome availability and pooling decisions, we compiled a consolidated summary table, Supplementary Materials File S3 (Table S8). This table lists, for each biomechanical outcome, the total number of studies contributing data, the number of studies contributing to each postoperative time window (<6 months, 6–<12 months, ≥12 months), and whether the outcome met the predefined pooling criterion (≥5 studies overall). Subgroup meta-analyses for postoperative time windows were only performed when the overall number of studies for that outcome reached this threshold, even if an individual time window contained fewer studies. Biomechanical outcomes reported in fewer than five studies were not included in any meta-analysis. If there were five or more studies overall but fewer than two studies for any of the time periods of interest (e.g., peak hip flexion angle, peak knee flexion moment, peak knee extension moment, and peak knee adduction moment), studies were categorized into two subgroups (less than 12 months and 12 months and over) else subgroup analysis was not performed.

### 2.6. Assessment of Publication Bias

Assessment of publication bias and potential asymmetry of the effect size was visually assessed using a contour-enhanced funnel plot after reviewing the meta-analytic data [37] (Supplementary Materials File S4: Figures S1–S6). The relationship between effect sizes and standard error was statistically verified using Egger’s regression to determine whether the funnel plot was asymmetric [38]. In the case of asymmetry, the mean effect size obtained by adjusting the asymmetry was calculated using the trim-and-fill method and compared with the original average effect size to determine the number of missing studies [39].

### 2.7. Statistical Analyses

Quantitative data synthesis was computed and illustrated in the form of forest plots using Cochrane Review Manager 5.1 (The Nordic Cochrane Centre, The Cochrane Collaboration, Copenhagen, Denmark). To examine the main research question, within-group paired-samples standardized mean differences (SMDs) with 95% confidence intervals (CI) were computed as effect size measures using a random-effects model to elucidate the effects of ACLR. Paired standardized mean differences were computed as Hedges’ g to correct for small-sample bias. All effects were calculated as ACLR minus contralateral limb, where negative values indicate reduced joint angles or moments in the ACLR limb. SMDs were categorized as trivial (0–0.2), small (0.2–0.5), moderate (0.5–0.8), and large (>0.8) [37,38,39]. Study heterogeneity was assessed using the I^2^ index. The level of heterogeneity was classified as high (>75%), moderate (50%–75%), and low (25%–50%) [40]. Publication bias in outcomes was assessed and treated using standard methodology. Funnel plots and Egger’s regression test were used to analyze publication bias.

## 3. Results

### 3.1. Results Related to Study Selection

**Figure 1:**
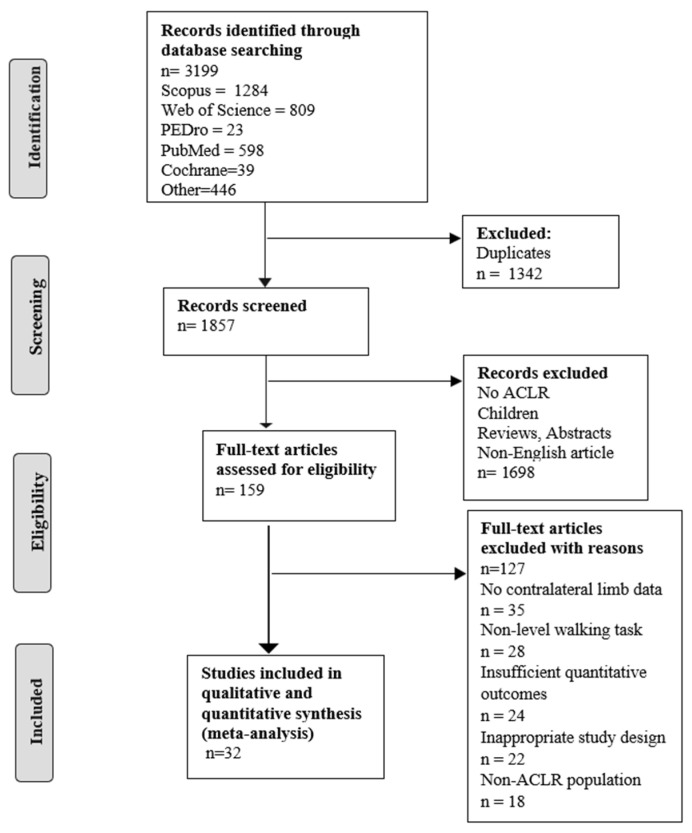
PRISMA flow chart of studies included in this systematic review with meta-analysis.

The initial search identified 3522 studies. After duplicate removal, 1857 studies remained. Following the screening of titles and abstracts, 159 full texts were further considered. Finally, quantitative analyses were computed with 32 articles. Thirty-two studies involving 1026 participants examined gait mechanics between the ACLR and the contralateral limb. Figure 1 presents a PRISMA flow chart and illustrates the study selection process. Meta-analyses were conducted on peak knee flexion angle, peak hip flexion angle, peak vertical ground reaction force, peak knee flexion moment, peak knee extension moment, and peak knee adduction moment variables. In the cases of peak hip flexion angle, peak knee flexion moment, peak knee extension moment, and peak knee adduction moment, outcome studies were categorized into two subgroups (less than 12 months and 12 months and over). For the peak vertical ground reaction force, subgroup analysis was not performed.

### 3.2. Study Characteristics

Thirty-two studies examined gait mechanics between the ACLR and contralateral limb (Table 2). Two studies did not provide information on the participants’ gender [40,41], three studies did not specify the type of graft used [42,43,44], and twenty studies did not report the time since injury. Five studies were classified into short-term [45,46,47,48,49], 10 studies into mid-term [8,13,24,25,40,49,50,51,52,53], and 20 studies into long-term post-surgery. Neal et al. [49] included both short-term and mid-term data, and two studies had both mid-term and long-term data [13,52]. All studies that analyzed kinetic variables used force plates. Only one study collected gait data on a treadmill [21], with all others using overground protocols. Graft types were mixed across most samples, and few studies reported graft-specific subgroups with extractable outcomes, preventing graft-type-specific analyses. Reporting of gait speed was highly variable. Although gait speed is an important functional indicator, only a portion of studies analyzed or controlled for speed differences. Out of 32 studies, 19 reported gait speed [20,23,24,40,41,54,55,56,57,58,59,60,61,62,63,64,65,66,67,68]. Participants in the studies of Goetschius et al., 2018 [21] walked at constant speeds on the treadmill. Kumar et al., 2018 [52] selected a fixed walking speed. The remaining studies mentioned that the participants walked at a self-selected comfortable pace, but did not analyze between-group differences [16,18,19,45,69,70,71,72].

### 3.3. Results Related to Quality Assessment

The methodological quality of the included 32 studies averaged 69% on the modified version of the Downs and Black checklist [34]. This is indicative of moderate methodological quality (Table 3). Among the 32 included studies, five were rated high quality [13,47,52,57,75] and four low quality [21,40,45,74]. Authors from the nine studies reported the calculation of a priori power analysis to estimate the sample size [42,43,44,50,53,57,72,73,74]. Inter-rater agreement was perfect for reporting items (κ = 0.84), substantial for confounding (κ = 0.69), and bias items (κ = 0.74).

### 3.4. Effects of ACLR on Lower Limb Joint Kinematics

**Figure 2:**
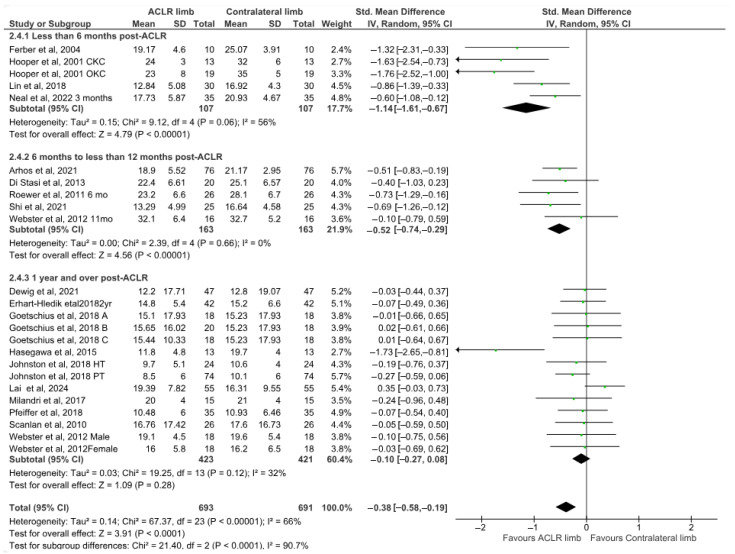
Forest plot illustrates the peak knee flexion angle difference between the ACLR and contralateral limb during walking [8,13,21,25,26,45,46,47,48,49,50,53,57,58,62,72,74,75,76]. The subgroup effect of the term since surgery for each parameter and the total effect were calculated as standardized mean difference (95% CI). Favors ACLR limb: lower in ACLR limb; Favors Contralateral limb: greater in ACLR limb; SD: Standard deviation; Std: Standardized; CI: Confidence interval.

Nineteen studies evaluated peak knee flexion angle differences between the ACLR and the contralateral limb during walking [8,13,21,25,26,45,46,47,48,49,50,53,57,58,62,72,74,75,76]. The findings indicated small but significant differences with moderate heterogeneity (19 studies: small SMDs = −0.39, 95% CI −0.58 to −0.19, *p* < 0.0001, I^2^ = 66%) (Figure 2). More specifically, the peak knee flexion angle was 2.63° (95% CI −3.81 to −1.44) lower in the ACLR compared to the contralateral limb. Based on the results of Egger’s test, there was no indication of publication bias (*p* = 0.12). The analysis of time post-surgery revealed significant differences in the short-term (four studies: large SMDs = −1.14, 95% CI −1.61 to −0.67, *p* < 0.00001, I^2^ = 56%) [45,47,48,49] and mid-term (five studies: small SMDs = −0.52, 95% CI −0.74 to −0.29, *p* < 0.0001, I^2^ = 0%) [8,13,25,50,53] after surgery but not for the long-term follow-up (10 studies: small SMDs = −0.10, 95% CI −0.27 to 0.07, *p* = 0.26, I^2^ = 32%) [21,26,46,57,58,62,72,74,75,76].

**Figure 3:**
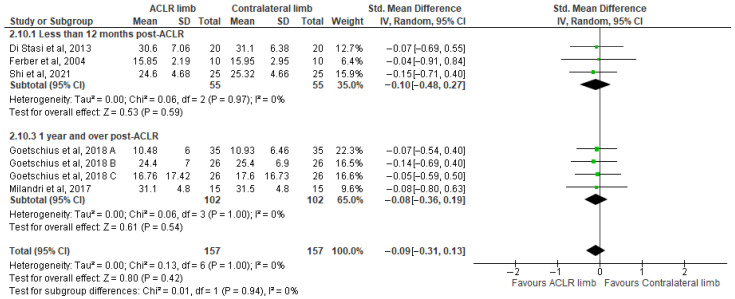
Forest plot illustrates the peak hip flexion angle difference between the ACLR and contralateral limb during walking [8,21,45,53,62]. The subgroup effect of the time since surgery for each parameter and the total effect were calculated as standardized mean difference (95% CI). Favors ACLR limb: lower in ACLR limb; Favors Contralateral limb: greater in ACLR limb; SD: Standard deviation; Std: Standardized; CI: Confidence interval.

Five studies evaluated peak hip flexion angle differences between the ACLR and the contralateral limb during walking [8,21,45,53,62]. Total and subgroup analysis indicated no evidence of study heterogeneity (I^2^ = 0%) and yielded no significant differences (five studies: trivial SMDs = −0.03, 95% CI −0.28 to 0.22, *p* = 0.82, I^2^ = 0%) (Figure 3). Based on the results of the Egger’s test, there was no indication of publication bias (*p* = 0.68).

### 3.5. Effects of ACLR on Lower Limb Kinetics

Vertical ground reaction force.

**Figure 4:**
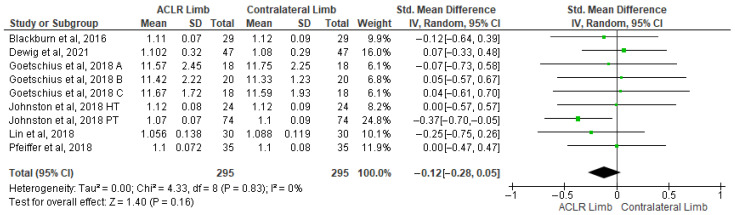
Forest plot illustrates the peak vertical ground reaction force difference in the ACLR and contralateral limb during walking [21,48,72,73,75]. The subgroup effect of the time since surgery for each parameter and the total effect were calculated as standardized mean difference (95% CI). Favors ACLR limb: lower in ACLR limb; Favors Contralateral limb: greater in ACLR limb; SD: Standard deviation; Std: Standardized; CI: Confidence interval.

Six studies evaluated peak vertical ground reaction force differences between the ACLR and the contralateral limb during walking [21,48,72,73,75]. The findings indicated no significant differences with no evidence of study heterogeneity (six studies: trivial SMDs = −0.12, 95% CI −0.28 to 0.05, *p* = 0.16, I^2^ = 0%) (Figure 4). Based on the results of the Egger’s test, there was no indication of publication bias (*p* = 0.57).

### 3.6. Knee Joint Moments

**Figure 5:**
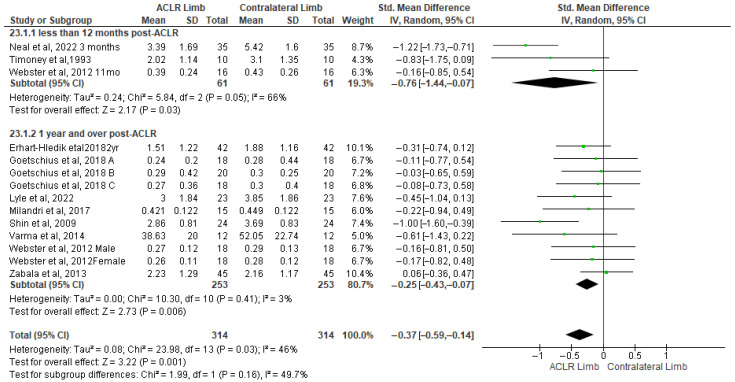
Forest plot illustrates the peak knee flexion moment difference in the ACLR and contralateral limb during walking [21,25,26,40,41,43,44,49,58,62,67]. The subgroup effect of the term since surgery for each parameter and the total effect were calculated as standardized mean difference (95% CI). Favors ACLR limb: lower in ACLR limb; Favors Contralateral limb: greater in ACLR limb; SD: Standard deviation; Std: Standardized; CI: Confidence interval.

Eleven studies evaluated peak knee flexion moment differences between the ACLR and the contralateral limb during walking [21,25,26,40,41,43,44,49,58,62,67]. The findings indicated a significantly small effect of ACLR with the low level of heterogeneity (11 studies: small SMDs = −0.37, 95% CI −0.59 to −0.14, *p* = 0.0001, I^2^ = 46%) (Figure 5). More specifically, the peak knee flexion moment was lower in the ACLR compared to the contralateral limb. Based on the results of the Egger’s test, there was no indication of publication bias (*p* = 0.12). The analysis of time post-surgery revealed a significantly lower peak knee flexion moment in the ACLR limb in less than 12 months (three studies: moderate SMDs = −0.76, 95% CI −1.44 to −0.07, *p* = 0.03, I^2^ = 66%) [25,26,40,49] and over 12 months (eight studies: small SMDs = −0.25, 95% CI −0.43 to −0.07, *p* = 0.01, I^2^ = 46%) [21,25,26,41,43,44,58,62,67] after surgery, compared to the contralateral limb.

**Figure 6:**
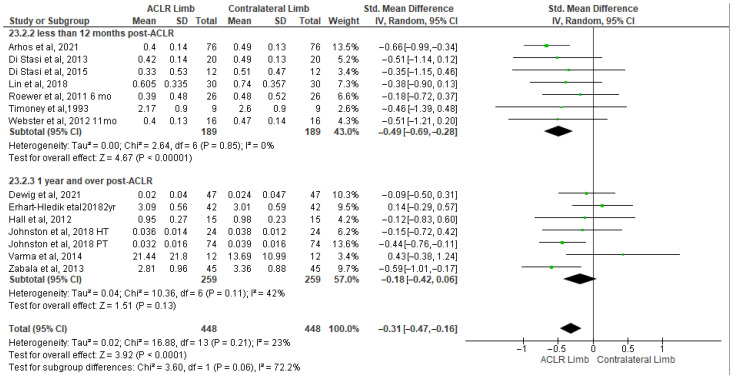
Forest plot illustrates the peak knee extension moment difference between the ACLR and contralateral limb during walking [8,13,25,40,41,48,50,51,57,58,67,69,72]. The subgroup effect of the term since surgery for each parameter and the total effect were calculated as standardized mean difference (95% CI). Favors ACLR limb: lower in ACLR limb; Favors Contralateral limb: greater in ACLR limb; SD: Standard deviation; Std: Standardized; CI: Confidence interval.

Thirteen studies evaluated peak knee extension moment differences between the ACLR and the contralateral limb during walking [8,13,25,40,41,48,50,51,57,58,67,69,72]. The findings indicated significantly small differences (13 studies: small SMDs = −0.31, 95% CI −0.47 to −0.16, *p* < 0.0001, I^2^ = 23%) (Figure 6). More specifically, the peak knee extension moment was lower in the ACLR compared to the contralateral limb. No evidence of study heterogeneity (I^2^ = 23%) was observed. Based on the results of the Egger’s test, there was no indication of publication bias (*p* = 0.35). The analysis of time post-surgery revealed a significantly lower peak knee extension moment in the ACLR limb in less than 12 months (seven studies: moderate SMDs = −0.49, 95% CI −0.69 to −0.28, *p* < 0.00001, I^2^ = 0%) [8,13,25,40,48,50,51] but not for over 12 months [41,57,58,67,69,72] (six studies: small SMDs = −0.18, 95% CI −0.42 to 0.06, *p* = 0.13, I2 = 42%).

**Figure 7:**
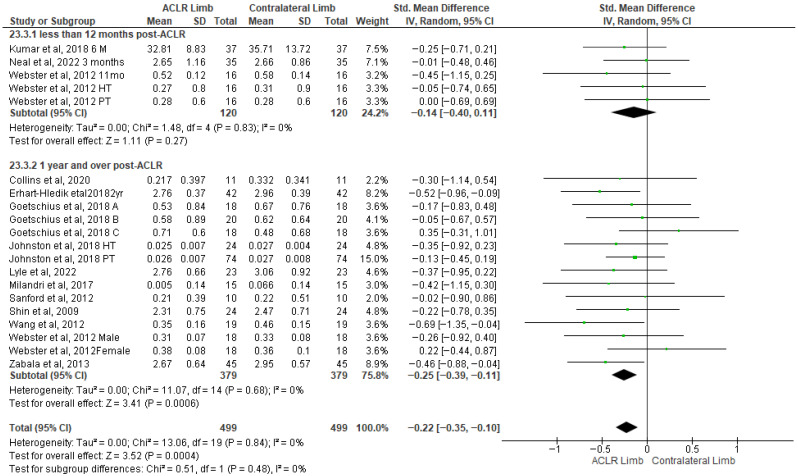
Forest plot illustrates the peak knee adduction moment difference between the ACLR and contralateral limb during walking [21,24,25,26,42,43,44,49,52,58,62,67,70,72,77]. The subgroup effect of the term since surgery for each parameter and the total effect were calculated as standardized mean difference (95% CI). Favors ACLR limb: lower in ACLR limb; Favors Contralateral limb: greater in ACLR limb; SD: Standard deviation; Std: Standardized; CI: Confidence interval.

Fourteen studies evaluated peak knee adduction moment differences between the ACLR and the contralateral limb during walking [21,24,25,26,42,43,44,49,52,58,62,67,70,72,77]. The findings indicated significant but small differences with no evidence of heterogeneity (14 studies: small SMDs = −0.22, 95% CI −0.35 to −0.10, *p* = 0.004, I^2^ = 0%) (Figure 7). More specifically, the peak knee adduction moment was lower in the ACLR compared to the contralateral limb. Based on the results of the Egger’s test, there was no indication of publication bias (*p* = 0.67). The analysis of time post-surgery revealed significantly lower peak knee extension moment in the ACLR limb over 12 months (10 studies: small SMDs = −0.25, 95% CI −0.39 to −0.11, *p* = 0.0006, I^2^ = 0%) [21,26,42,43,44,58,62,65,67,70,72] but not for less than 12 months (four studies: small SMDs = −0.14, 95% CI −0.40 to 0.11, *p* = 0.27, I^2^ = 0%) after surgery walking [24,25,49,52].

## 4. Discussion

This systematic review with meta-analysis aimed to compare the kinematic (e.g., knee flexion motion) and kinetic (e.g., knee moment and ground reaction forces) variables of the involved limb with the contralateral limb in individuals who had undergone an ACLR from short-term to mid-term and long-term periods after surgery during walking in ACLR knees. The peak knee flexion angle was lower in the ACLR limb when compared to the contralateral limb, with significant differences observed in the short-term and mid-term postoperative periods. Based on findings of the present meta-analyses, it can be hypothesized that surgical treatment results in altered biomechanical gait characteristics, such as peak knee flexion moment, that persist ≥12 months post-surgery. These surgery-related biomechanical changes in gait characteristics may constitute a risk factor for injury and also knee osteoarthritis in ACLR patients [78].

Regarding kinetic variables, the peak knee flexion moment, peak knee adduction moment, and peak knee extension moment were lower in the ACLR limb compared to the contralateral limb. More specifically, the peak knee flexion angle was 2.6° lower in the ACLR compared to the contralateral limb. However, the observed difference appears not to be clinically meaningful. While we observed a knee flexion difference of 2.6° between ACLR compared to the contralateral limb, clinical meaningfulness appears to be given to a knee flexion difference of 3° [62,79]. Neurophysiological changes following ACL injury and reconstruction have been shown to alter lower limb muscle activation [80] and lower cortical activation in injured limbs [81]. The presence of pain, reported even at 2–5 years after surgery [82], or reflex inhibition [83], could further influence the movement patterns, as apparent with reduced flexion moments. Furthermore, compensatory movements at the hip may also decrease knee joint loading [84]. It is thus likely that multiple mechanisms contribute to long-term decreased flexion moments in the ACLR knees. Subgroup analyses revealed a significantly reduced peak knee flexion moment in the ACLR limb within less than 12 months and over 12 months post-surgery. Furthermore, the peak knee extension moment was lower in the ACLR limb during less than 12 months, while the peak knee adduction moment was diminished in the ACLR limb over 12 months post-surgery compared to the contralateral limb.

A great deal of asymmetry following surgery is also brought into focus by the comparison of the ACLR limb and its contralateral non-operated limb. The data indicate that the ACLR limb consistently demonstrated reduced knee flexion, though this difference gradually diminished over the short-, mid-, and long-term post-surgery periods. It is consistent with ACLR limb movements where great loads are applied both for extending and flexing knees around the region. A reduced knee flexion angle following ACL reconstruction is clinically concerning, as it may redistribute mechanical loads to cartilage regions that are structurally unprepared for the altered stress environment post-surgery [85]. From 0 to 30° of knee flexion, the contact point in the medial compartment shifts posteriorly as the knee flexion angle increases [86,87,88]. This suggests that individuals with reduced knee flexion angles may place greater loads on more anterior cartilage regions, which are typically thinner and less equipped to withstand such stresses [88,89,90]. Altered stress distribution in these newly loaded regions may initiate irreversible cartilage damage [90]. The peak knee flexion angle of the involved limb typically increases up to one year after ACLR, with no significant differences between limbs reported beyond this timeframe [30,91]. A new concern arises when the point of contact within the joint now moves posteriorly as these flexion angles increase, as this region of cartilage may have experienced lower loads and stresses since ACLR [88,90,92]. During this period of reduced loading, the cartilage may undergo adaptations resembling non-weight-bearing cartilage, potentially compromising its ability to withstand the return to pre-injury load levels [90,92]. Therefore, irreversible cartilage damage after ACLR may result not only from the reduction in knee flexion angle but also from the recovery of range of motion over the one-year postoperative period. Further studies are needed to investigate this hypothesis and to determine what constitutes a clinically meaningful change in knee flexion angle throughout the stance phase.

Some limitations may affect the results of this study. Force-plate use did not vary; only one study used treadmill walking [21]; graft types were inconsistently reported; and gait speed protocols were heterogeneous and often not statistically examined. These limitations reduced our ability to evaluate whether methodological or clinical factors contributed to between-study heterogeneity. Previous studies showed distinct biomechanical characteristics for females and males. Asaeda et al., 2017 [16], identified sex-based differences in tibial rotation that influenced the kinematics and kinetics of the knee over the stance phase of gait, both pre-operatively and post-ACLR. In our meta-analysis, we included studies with participants of mixed sexes. Webster et al. [23], examined the secondary planes of movement during walking in the ACLR participants to evaluate the influences of graft type (hamstring or patellar tendon). They revealed that reduced knee varus in the hamstring group may relate to the graft harvest. However, graft type was not considered in this analysis. Also, studies had different times since injury, which may have affected the results of this meta-analysis. Nonetheless, the time elapsed since injury was not accounted for in this analysis.

## 5. Conclusions

In conclusion, this systematic review with meta-analysis demonstrated that patients with ACLR exhibit persistent biomechanical asymmetries during walking, characterized by reduced peak knee flexion angles in the short- and mid-term postoperative periods, though these differences normalized in the long-term. Kinetically, the ACLR limb showed diminished peak knee flexion moments at all times, while peak knee extension moments were reduced only in the less than 12-month period and peak adduction moments only in the more than 12-month post-surgery period compared to the contralateral limb. These findings suggest a time-dependent compensatory mechanism, where protective adaptations (e.g., reduced flexion/extension moments) may initially offload the reconstructed limb, with some asymmetries resolving over time. The persistence of reduced flexion and adduction moments highlights potential long-term neuromuscular or biomechanical deficits that could influence joint health. Clinically, these results underscore the need for rehabilitation strategies tailored to address phase-specific deficits, promoting symmetrical loading and functional recovery. Further research should investigate the mechanisms driving these temporal patterns and their implications for osteoarthritis risk and functional outcomes.

## Figures and Tables

**Table 1:** Adapted PECOS (participants, exposure, comparators, outcomes, and study design) framework for study inclusion and exclusion criteria.

Category	Inclusion	Exclusion
**Participants**	Male and female individuals with ACLR (any type of graft) after isolated ACL injury or in addition to meniscus and/or collateral ligament injury, age greater than 18 years old	Individuals with adverse health events (e.g., injuries, recent surgery); individuals with neurological, systemic, or degenerative conditions
**Exposure**	ACL surgery	-
**Comparator**	Comparison with the contralateral limb	Absence of contralateral limb data
**Outcome**	Lower limb kinematics and kinetics during walking	Measures of lower limb kinematics and kinetics during activities other than walking (e.g., running, jumping); no measures of lower limb kinematics and kinetics
**Study design**	Case control studies, case series, cross-sectional studies, randomized controlled trials (baseline), cohort studies.	Case studies, systematic reviews

**Table 2:** 32 studies for quantitative analyses of ACLR limb vs. healthy limb.

Authors, Year	Number of ACLR Participants, Sex, Age, Year (Mean ± SD or Range)	Graft Type	Time Since Surgery	Time Since Injury	Walking Protocol	Outcomes	Kinematic and Kinetic Outcomes
Arhos et al., 2021 [50]	76, 38 M/38 F, 21.2 ± 7.7	17 allograft, 22 bone–patellar tendon–bone, 37 Hamstring	7.1 ± 2.0 months	NR	3 trials, self-selected speed, walkway	peak knee flexion angle, peak internal knee extension moment, and sagittal plane knee excursion	3D, force plate
Blackburn et al., 2016 [73]	29, F, 21.7 ± 3.1	20 patellar tendon; 9 hamstring tendon	47 ± 41.7 months	48 ± 41 months	5 trials, self-selected speed	vGRF	Force plate
Collins et al., 2020 [42]	11, 8M/3F, 20.55 ± 1.86	NR	1 year	44.95 ± 25.25 months	3 trials, 1.4 m/s, 5-m walkway	Peak knee adduction moment	3D, force plate
Dewig et al., 2021 [57]	47, 13 M/34 F, 20.8 ± 3.2	23 patellar tendon grafts, 22 hamstring tendon grafts, 1 quadriceps tendon graft, 1 allograft;	4 ± 3 years	NR	5 trials, 6 m walking	Kinetics (peak knee extension moment, knee abduction moment, vGRF) and kinematic (peak knee flexion angle and knee flexion displacement)	3D, force plate
Di Stasi et al., 2013 [8]	42 30 M/12 F, 29.3 ± 10.8, pass: 20, 25.4 ± 9.9;	Soft tissue allograft or a semitendinosus/gracilis autograft	6 months	Pass: 12.9 ± 7.1 weeks;	5 trials, walkway	Knee kinematics and kinetics	3D, force plate
Di Stasi et al., 2015 [51]	Male: 27, 28 ± 10; Female: 12, 32 ± 12	Hamstring autograft or soft tissue allograft	6 months	Male: 12.0 ± 10.0 Female: 9.0 ± 10.3	13 m walkway, self-selected speed	Peak knee flexion angle and peak knee extension angle	3D
Erhart-Hledik et al., 2018 [58]	2-year follow-up: 42, 29.6 ± 7.3; 8-year follow-up: 17, 5M/12 F, 35.1 ± 7.3	15 Achilles allograft, 1 bone–patellar tendon–bone allograft, and 1 bone–patellar tendon–bone autograft	7.7 ± 0.7 years and 2.2 ± 0.3 years	2.0 ± 1.8 months	3 trials, self-selected speed, 10 m walkway	Knee kinematics and kinetics	3D, force plate
Ferber et al., 2004 [45]	10, 5 M/5 F, 27.7 ± 9.1	Bone–patellar–bone	3 months	5:7 ± 5.1 years	12 trials, 5 m wooden walkway	Hip and knee joint moments, powers, and positions	3D, force plate
Goetschius et al., 2018 [21]	A: early ACLR: 18, 7 M/11 F, 21.7 ± 4.1; B: mid ACLR: 20, 4 M/16 F, 20.5 ± 2.2; C: late ACLR: 18, 6 M/12 F, 26.7 ± 4.4	Early ACLR: 7 Patella tendon, 10 Hamstring, and 1 Cadaver; mid ACLR: 11 Patella tendon, 5 Hamstrings, and 4 Cadaver; late ACLR: 10 Patella tendon, 5 Hamstrings, and 3 Cadaver.	Early ACLR: 1.4 ± 0.4 year; mid ACLR: 3.3 ± 0.6 years; late ACLR: 8.6 ± 2.8 years	NR	10 strides for each limb, at standardized speeds of 1.34 m/s on treadmill	Knee and hip kinetics and kinematics in the sagittal and frontal planes and vGRF	3D, treadmill force plate
Hall et al., 2012 [69]	15, 7 M/8 F, 26 ± 6	41% hamstring graft, 41% patellar tendon, 12% combination of cadaver and hamstring, and 6% cadaver	6 years	NR	6 trials,10 m walkway, self-selected speeds	Knee flexion angle, maximum internal ankle plantar flexion, knee extension, hip extension, hip abduction, and external knee varus moments	3D, force plate
Hasegawa et al., 2015 [46]	13, 7 M/6 F, 22.8	Semitendinosus and gracilis tendons	3 months	NR	2 trials, self-selected speed, 10 m walkway	Knee flexion angle, abduction angle, and tibial rotation angle	3D, force platform
Hooper et al., 2001 [47]	Closed Kinetic Chain (CKC): 13 M, 5 F; Open Kinetic Chain (OKC):19, 16 M, 3 F	Bone–patellar tendon–bone	2 weeks	CKC: 50.3 ± 61.8 months OKC: 34.1 ± 30.4 months	10 m walkway	Knee joint angle, moment, and power	3D, force platform
Johnston et al., 2018 [72]	PT: 74, 29 M/45 F, 22 ± 3; HT: 24, 5 M/19 F, 21 ± 4	74 Patella tendon and 24 Hamstring	PT: 22 ± 26 months; HT: 43 ± 34 months	NR	5 trials, walkway, self-selected speed	Knee joint angles, peak vGRF, peak instantaneous vGRF loading rate (first time derivative), and peak internal knee extension and valgus moments	3D, force platform
Kumar et al., 2018 [52]	37, 22 M/15 F, 30.8 ± 5.3	27 semitendinosus autograft, 8 tibialis posterior allograft, 1 patellar tendon allograft, 1 semitendinosus allograft	6 and 12 months	NR	4 trials, walked over-ground at a pace of 1.35 m/s	Peak knee adduction angle, knee adduction moment impulse, peak knee adduction moment	3D, force platform
Lai et al., 2024 [74]	55, 48 M/7 F, 30.6 ± 6.4	Semitendinosus/gracilis autograft	22.7 ± 20.0 months	NR	3.0 ± 0.3 km/h	Flexion/extension angle	3D
Lin et al., 2018 [48]	30, 10 M/20 F, 28 ± 12	14 Bone–Patellar Tendon–Bone autograft, 3 Hamstring autograft, 2 Quad tendon autograft, 11 Allograft	108 ± 17 days	NR	4 trials, self-selected, 10 m path	Peak knee extensor moment, peak vertical and posterior ground reaction force, and peak anterior shank angular velocity	3D, force platform
Lyle et al., 2022 [43]	23, 13 M/10 F, 25.1 ± 6.3	NR	14.4 ± 17.2 months	NR	3 trials, self-selected speed (1.37 ± 0.17 m/s)	Peak knee-flexion and adduction moments	3D, force platform
Milandri et al., 2017 [62]	15, M, 37.4 ± 10.7	Hamstring autograft	4 and 6 years	NR	5 trials, walkway, self-selected speeds	Hip and knee flexion angles, external hip and knee flexion moments, external knee adduction moment, and knee varus	3D, force platform
Neal et al., 2022 [49]	35, 17 M/18 F, 22 ± 6	6 soft-tissue allograft, 11 hamstring autograft, 18 bone–patellar tendon–bone autograft	3.2 ± 0.5 and 6.4 ± 0.7 months	NR	Self-selected speeds 1.6 ± 0.2 m/s	Knee gait variables (flexion angle, flexion moment, adduction moment, extensor forces, and medial compartment force)	3D, force platform
Pfeiffer et al., 2018 [75]	35, 26 M/9 F,	26 patellar tendon autograft and 9 semitendinosus/gracilis autograft	49.65 months	NR	5 trials, 6 m walkway, self-selected speed	Peak knee flexion angle, peak knee adduction angle and displacement, and peak knee abduction angle and displacement, vGRF peak magnitude, instantaneous and linear loading rates, peak internal knee extension moment, and peak internal knee abduction moments	3D, force platform
Roewer et al., 2011 [13]	26, 18 M/8 F, 29.6 ± 10.7	Semitendinosis-gracilis autograft or a soft tissue allograft	6 months and 2 years	NR	5 trials, 13 m walkway, speed of 1.56 ± 0.11 m/s	Peak knee flexion angle and knee joint excursion during weight acceptance, internal knee and hip extensor moments at peak knee flexion, and peak knee power absorption	3D, force platform
Sanford et al., 2012 [70]	10, 4 M/6 F	Bone–patellar tendon–bone graft or 1 hamstring tendon	7.4 ± 5.8 years	NR	3 trials, 10 m walkway, self-selected pace	The knee flexion, adduction, and internal rotation moments	3D, force platform
Scanlan et al., 2010 [76]	26, 11 M/15 F, 31	12 allografts (9 Achilles, 1 bone–patellar–bone, and 2 soft tissue allograft) and 14 autografts (10 bone–patellar–bone and 4 hamstrings tendons)	24 months	NR	3 trials, self-selected speed, walkway	Internal–external rotation, varus–valgus, and knee flexion	3D
Shi et al., 2021 [53]	25, M, 32.0 ± 8.2	Hamstring tendon autograft	7.4 ± 1.3 months	NR	3 trials, 10 m walkway at self-selected walking speed	Peak knee and hip flexion	3D
Shin et al., 2009 [44]	24, 9 M/15 F, 33.6 ± 9.2	NR	34 ± 34 months	43 ± 38 months	3 trials, self-selected speed, walkway	Flexion moment, Adduction moment, Abduction moment, Internal rotation moment, External rotation moment	3D, force platform
Timoney et al., 1993 [40]	10, 20, and 30 years	Patellar tendon	10 months (range, 9 to 12).	30 months with a range of 1 to 66 months	Walkway	Knee flexion moment, external knee moment	3D, force platform
Varma et al., 2014 [41]	12, 30.5 ± 8.68	Single bundle hamstring autograft	4.5 (3.5) years	NR	3 trials, 7 m walkway, self-selected pace	Peak knee flexion and adduction moments	3D, force platform
Wang et al., 2012 [77]	ACLR on the dominant side (dACLR): 19, 12 M/7 F, 32.4 ± 8.6 Non-dominant side (nACLR): 22, 12 M/10 F, 31.1 ± 8.0	3 Bone–patella tendon–bone graft, 16 Hamstring tendon graft	dACLR: 14.1 (4.4) months; nACLR: 13.9 ± 5.5	NR	10 trials, self-selected speeds	Knee adduction moment	3D, force plate
Webster et al., 2012 [26]	18, F, 27.7 ± 6.8; 18 M, 27.4 ± 6.6	Four-strand hamstring autograft	Female: 19.8 ± 11.6 months; male: 20.3 ± 11.4 months	Female: 13.1 ± 9.0 weeks; Male:14.8 ± 10.0 weeks	Self-selected speeds, 10 m Walkway	Maximum flexion and adduction angles and moments at the knee	3D, force plate
Webster et al., 2012 [24]	patellar tendon: 16, M, 23.8 ± 6; hamstring tendon: 16, M, 27.5 ± 6	16 patellar tendon and 16 hamstring tendon	Patellar tendon: 11.2 ± 2 months; hamstring tendon: 9.4 ± 3 months	Patellar tendon: 11.9 ± 11 hamstring tendon: 10.7 ± 9	3 trials, comfortable-speed, walkway	Knee adduction moment, knee varus angle, and vGRF	3D, force platform
Webster et al., 2012 [25]	16, 13 M/3 F, 26 ± 6	8 hamstring graft, 8 bone–patellar–tendon bone	10 ± 2 months and 3.3 ± 0.4 years	NR	≥3 trials, self-selected speed	Knee flexion–extension, varus–valgus, and internal–external rotation angle	3D, force plate
Zabala et al., 2013 [67]	45, 26 M/19 F, 29.5 ± 6.1	Achilles allograft	26 months	2.2 months	3 trials, comfortable-speed, Walkway	Knee ad/abduction moment, flexion/extension moment, and external/internal rotation moment	3D, force platform

ACLR: anterior cruciate ligament reconstruction, vGRF: Vertical ground reaction forces, ROM: Range of motion, 2D: 2-dimensional; 3D: 3-dimensional; NR: Not Reported; SD: standard deviation, M: male, F: female.

**Table 3:** Downs and Black methodological quality assessment scores of the 32 included studies.

Studies	Reporting								External Validity		Internal Validity (Bias)				Internal Validity (Confounding)			Power	Score (%)	Quality
	1	2	3	4	5	6	7	10	11	12	15	16	18	20	21	22	25	27		
**Arhos et al., 2021** [50]	1	1	1	1	2	1	1	1	0	0	0	1	1	1	0	0	1	1	74	MODERATE
**Blackburn et al., 2016** [73]	1	1	1	1	2	1	1	1	0	0	0	1	1	1	0	0	1	1	74	MODERATE
**Collins et al., 2020** [42]	1	1	1	1	2	1	1	1	0	0	0	1	1	1	0	0	1	1	74	MODERATE
**Dewig et al., 2021** [57]	1	1	1	1	2	1	1	1	0	0	0	1	1	1	0	1	1	1	79	HIGH
**Di Stasi et al., 2013** [8]	1	1	1	1	2	1	1	1	0	0	0	1	1	1	0	0	1	0	68	MODERATE
**Di Stasi et al., 2015** [51]	1	1	1	1	2	1	1	1	0	0	0	1	1	1	0	0	1	0	68	MODERATE
**Erhart-Hledik et al., 2018** [58]	1	1	1	1	2	1	1	1	0	0	0	1	1	1	0	0	1	0	68	MODERATE
**Ferber et al., 2004** [45]	1	1	1	1	1	1	1	0	0	0	0	1	1	1	0	0	1	0	58	LOW
**Goetschius et al., 2018** [21]	1	1	1	1	1	1	1	0	0	0	0	1	1	1		0	1	0	58	LOW
**Hadizadeh et al., 2016** [60]	1	1	1	1	1	1	1	1	0	0	0	1	1	1	0	0	1	0	63	MODERATE
**Hall et al., 2012** [69]	1	1	1	1	2	1	1	1	1	0	0	1	1	1	0	0	1	0	74	MODERATE
**Hasegawa et al., 2015** [46]	1	1	1	1	1	1	1	1	0	0	0	1	1	1	0	0	1	0	63	MODERATE
**Hooper et al., 2001** [47]	1	1	1	1	2	1	1	1	1	0	0	1	1	1	1	0	1	0	79	HIGH
**Johnston et al., 2018** [72]	1	1	1	1	2	1	1	1	0	0	0	1	1	1	0	0	1	1	74	MODERATE
**Kumar et al., 2018** [52]	1	1	1	1	2	1	1	1	1	0	0	1	1	1	1	0	1	0	79	HIGH
**Lai et al., 2024** [74]	1	1	1	1	1	1	0	0	0	0	0	1	1	1	0	0	1	1	58	LOW
**Lin et al., 2018** [48]	1	1	1	1	2	1	1	1	0	0	0	1	1	1	0	0	1	0	68	MODERATE
**Lyle et al., 2022** [43]	1	1	1	1	2	1	1	1	0	0	0	1	1	1	0	0	1	1	74	MODERATE
**Milandri et al., 2017** [62]	1	1	1	1	2	1	1	1	0	0	0	1	1	1	0	0	1	0	68	MODERATE
**Neal et al., 2022** [49]	1	1	1	1	2	1	1	1	0	0	0	1	1	1	0	0	1	0	68	MODERATE
**Pfeiffer et al., 2018** [75]	1	1	1	1	2	1	1	1	1	0	0	1	1	1	1	0	1	0	79	HIGH
**Roewer et al., 2011** [13]	1	1	1	1	2	1	1	1	1	0	0	1	1	1	1	0	1	0	79	HIGH
**Sanford et al., 2012** [70]	1	1	1	1	2	1	1	1	0	0	0	1	1	1	0	0	1	0	68	MODERATE
**Scanlan et al., 2010** [76]	1	1	1	1	2	1	1	1	0	0	0	1	1	1	0	0	1	0	68	MODERATE
**Shi et al., 2021** [53]	1	1	1	1	2	1	1	1	0	0	0	1	1	1	0	0	1	1	74	MODERATE
**Shin et al., 2009** [44]	1	1	1	1	1	1	1	1	0	0	0	1	1	1	0	0	1	1	68	MODERATE
**Timoney et al.,1993** [40]	0	1	1	1	1	1	1	0	0	0	0	1	1	1	0	0	1	0	53	LOW
**Varma et al., 2014** [41]	1	1	1	1	2	1	1	1	0	0	0	1	1	1	0	0	1	0	68	MODERATE
**Wang et al., 2012** [77]	1	1	1	1	2	1	1	1	0	0	0	1	1	1	0	0	1	0	68	MODERATE
**Webster et al., 2012** [25]	1	1	1	1	1	1	1	1	0	0	0	1	1	1	0	0	1	0	63	MODERATE
**Webster et al., 2012** [24]	1	1	1	1	1	1	1	1	0	0	0	1	1	1	0	0	1	0	63	MODERATE
**Webster et al., 2012** [26]	1	1	1	1	1	1	1	1	0	0	0	1	1	1	0	0	1	0	63	MODERATE
**Zabala et al., 2013** [67]	1	1	1	1	1	1	1	1	0	0	0	1	1	1	0	0	1	0	63	MODERATE
**Average score (mean (SD))**																			69	MODERATE

1 = Yes; 0 = No; SD: Standard Deviation; High Quality (Score ≥ 75%); Moderate Quality (60% ≤ Score < 75%); Low Quality (Score < 60%).

## Data Availability

No new data were created or analyzed in this study.

## Supplementary Materials

The following supporting information can be downloaded at: https://www.mdpi.com/article/10.3390/healthcare13243304/s1, File S1: Search syntax in different electronic databases: Table S1: Search syntax for PubMed from inception until 13 November 2025; Table S2: Search syntax for Physiotherapy Evidence Database (PEDro) from inception until 15 November 2025; Table S3: Search syntax for Scopus from inception until 25 November 2025; Table S4: Search syntax for Web of Science from inception until 14 November 2025; Table S5: Search syntax for Cochrane Central Register of Controlled Trials (central) from inception until 15 November 2025; File S2: Risk of Bias Assessment: Modified Downs and& Black Checklist: Table S6: Modified Downs and& Black items used in this review; Table S7: Operational Definitions for Each Item; File S3: Summary of studies and time windows per outcome: Table S8: Number of studies contributing to each outcome and each postoperative time window; File S4: Funnel plots indicating potential publication bias: Figure S1: Funnel plot indicating potential publication bias for the parameter peak knee adduction angle; Figure S2: Funnel plot indicating potential publication bias for the parameter peak hip flexion angle; Figure S3: Funnel plot indicating potential publication bias for the parameter peak vertical ground reaction force; Figure S4: Funnel plot indicating potential publication bias for the parameter peak knee flexion moment; Figure S5: Funnel plot indicating potential publication bias for the parameter peak knee extension moment; Figure S6: Funnel plot indicating potential publication bias for the parameter peak knee adduction moment.

## Abbreviations

The following abbreviations are used in this manuscript:

ACLR	Anterior Cruciate Ligament Reconstruction
SMDs	Standardized Mean Differences
CI	Confidence Intervals
ACL	Anterior Cruciate Ligament
CENTRAL	Cochrane Central Register of Controlled Trials
vGRF	Vertical Ground Reaction Forces
ROM	Range of Motion
2D	2-Dimensional
3D	3-Dimensional
NR	Not Reported
SD	Standard Deviation
M	Male
F	Female
