# Supplementary material for: Gait Biomechanical Differences in the Anterior Cruciate Ligament Reconstructed and Contralateral Limb: A Systematic Review with Meta-Analysis
Source: Healthcare (Basel). 2025 Dec 16;13(24):3304. doi: 10.3390/healthcare13243304 (PMC12733360; doi:10.3390/healthcare13243304)
Supplement: Supplementary file 1 [file healthcare-13-03304-s001.zip › healthcare-3976506-supplementary.pdf]

Supplementary Material File S1: Search syntax in different electronic databases

Table S1: Search syntax for PubMed from inception until November 13, 2025.

Search number	Query
15	#2 AND #8 AND #14
14	#11 OR #12 OR #13
13	"Lower Extremity"[Mesh] OR "Lower Extremity Deformities, Congenital"[Mesh] OR "Foot"[Mesh] "Foot Joints"[Mesh] OR "Ankle Joint"[Mesh] OR "Ankle"[Mesh] OR "Knee Joint"[Mesh] OR "Knee"[Mesh] OR "Hip Joint"[Mesh] OR "Hip"[Mesh] OR "Pelvis"[Mesh] OR "Thigh"[Mesh]
12	"Lower limb*" [ti] OR "lower extremit*" [ti] OR foot [ti] OR feet [ti] OR ankle [ti] OR ankles [ti] OR leg [ti] OR legs [ti] OR knee [ti] OR knee* [ti] OR hip [ti] OR hips [ti] OR pelvis [ti] OR thigh [ti] OR thighs [ti]
11	"Lower limb*" [tiab] OR "lower extremit*" [tiab] OR foot [tiab] OR feet [tiab] OR ankle [tiab] OR ankles [tiab] OR leg [tiab] OR legs [tiab] OR knee [tiab] OR knee* [tiab] OR hip [tiab] OR hips [tiab] OR pelvis [tiab] OR thigh [tiab] OR thighs [tiab]
9	#4 AND #8
8	#5 OR #6 OR #7
7	biomechanic* [ti] OR kinematic* [ti] OR motion* [ti] OR movement* [ti] OR pressure* [ti] OR dynamic [ti] OR load* [ti] OR biomech* [ti] OR mechanic* [ti] OR shock* [ti] OR absorb* [ti] OR friction* [ti] OR moment* [ti] OR angle* [ti] OR rotation* [ti] OR force* [ti] OR "angular impuls*" [ti] OR velocit* [ti] OR speed* [ti] OR acceleration* [ti] OR muscle* [ti] OR activit* [ti] OR torque* [ti] OR power* [ti]
6	(friction* [tiab] OR moment* [tiab] OR angle* [tiab] OR rotation* [tiab] OR force* [tiab] OR angular* [tiab] Or impuls* [tiab] OR velocit* [tiab] OR speed* [tiab] OR acceleration* [tiab] OR activit* [tiab] OR mechanic* [tiab] OR power* [tiab] OR biomechanic* [tiab] OR kinematic* [tiab] OR motion* [tiab] OR movement* [tiab] OR pressure* [tiab] OR dynamic* [tiab] OR load* [tiab] OR biomech* [tiab] OR mechanic* [tiab] OR shock* [tiab] OR absorb* [tiab])
5	"biomechanical phenomena"[Mesh] OR "mechanical phenomena"[Mesh]
4	#1 OR #2 OR #3
3	“anterior cruciate ligament reconstruction” [tiab] OR "anterior cruciate ligament" [tiab] OR "anterior cruciate ligament injur*" [tiab] OR “ACL repair” [tiab]
2	"anterior cruciate ligament" [ti] OR "anterior cruciate ligament injur*" [ti] OR “ACL repair” [ti]
1	"anterior Cruciate Ligament"[Mesh] OR "anterior cruciate ligament reconstruction"[Mesh] OR "anterior cruciate ligament injur*"[Mesh]

Search results based on PubMed 598

Table S2: Search syntax for Physiotherapy Evidence Database (PEDro) from inception until November 15, 2025.

First search	Second search	Third search
• Abstract & title: anterior cruciate ligament* AND	• Abstract & title: anterior cruciate ligament* AND	• Abstract & title: anterior cruciate ligament* AND
• Body part: foot and ankle AND	• Body part: lower leg and knee AND	• Body part: thigh or hip AND
• Method: clinical trial	• Method: clinical trial	• Method: clinical trial

Search results based on PEDro: 346

Table S3: Search syntax for Scopus from inception until November 25, 2025.

Participants	AND	Task	AND	Outcomes	AND	Body part
"anterior cruciate ligament reconstruction"		walk*		biomech*		"lower limb*"
OR "anterior cruciate ligament"		OR walking*		OR kinetic*		OR "lower extremit*"
OR "anterior cruciate ligament injur*"		OR gait*		OR kinematic*		OR foot
OR "ACL repair"		OR "translation movement"		OR speed		OR feet
				OR force*		OR ankle
				OR motion*		OR ankles
				OR rotation*		OR leg
				OR impuls		OR legs
				OR acceleration		OR knee
				OR dynamic		OR knee*
				OR power		OR hip
				OR movement		OR hips
				OR load*		OR pelvis

		OR joint moment*	OR thigh*
		OR ground reaction force*	
		OR mechanic*	
		OR torque*	
		OR angle*	

1,284 results from Scopus

Table S4: Search syntax for Web of Science from inception until November 14, 2025.

Participants	AND	Task	AND	Outcomes	AND	Body part
"anterior cruciate ligament reconstruction"		walk*		biomech*		"lower limb*"
OR "anterior cruciate ligament"		OR walking*		OR kinetic*		OR "lower extremit*"
OR "anterior cruciate ligament injur*"		OR gait*		OR kinematic*		OR foot
OR "ACL repair"		OR "translation movement"		OR speed		OR feet
				OR force*		OR ankle
				OR motion*		OR ankles
				OR rotation*		OR leg
				OR impuls		OR legs
				OR acceleration		OR knee
				OR dynamic		OR knee*
				OR power		OR hip
				OR movement		OR hips
				OR load*		OR pelvis
				OR joint moment*		OR thigh*
				OR ground reaction force*		
				OR mechanic*		
				OR torque*		
				OR angle*		

809 results from Web of Science core collection

Table S5: Search syntax for Cochrane Central Register of Controlled Trials (central) from inception until November 15, 2025.

ID	Search
**#1**	MeSH descriptor: [anterior cruciate ligament] explode all trees
**#2**	MeSH descriptor: [biomechanical phenomena] explode all trees
**#3**	biomech*
**#4**	kinetic*
**#5**	kinematic*
**#6**	speed
**#7**	force*
**#8**	motion*
**#9**	rotation*
**#10**	impulse
**#11**	acceleration
**#12**	dynamic
**#13**	power
**#14**	movement
**#15**	load*
**#16**	joint moment*
**#17**	ground reaction force*
**#18**	mechanic*
**#19**	torque*
**#20**	angle*
**#21**	#2 OR #3 OR #4 OR #5 OR #6 OR #7 OR #8 OR #9 OR #10 OR #11 OR #12 OR #13 OR #14 OR #15 OR #16 OR #17 OR #18 OR #19 OR #20

**#22**	MeSH descriptor: [Lower Extremity] explode all trees
**#23**	#1 AND #21 AND #22

Total of 39 hits for Cochrane Central Register

Supplementary Material File S2: Risk of Bias Assessment: Modified Downs and Black Checklist

Table S6. Modified Downs and Black items used in this review

Item No.	Item (Modified Wording)	Domain
1	Objectives clearly described	Reporting
2	Main outcomes clearly defined	Reporting
3	Participant characteristics clearly described	Reporting
4	Interventions/conditions clearly described (ACLR limb vs contralateral limb protocols)	Reporting

5	Principal confounders clearly described (e.g., walking speed, graft type, concomitant injuries)	Reporting/Confounding
6	Main findings clearly described	Reporting
7	Estimates of random variability provided (SD/SE/CI)	Reporting
10	Actual probability values reported (not only $p<0.05$)	Reporting
11	Participants representative of the source population	External validity
12	Recruitment representative of the broader ACLR population	External validity
15	Data collected prospectively	Internal validity — bias
16	Participants blinded to research question (if applicable)	Internal validity — bias
18	Outcome assessors blinded to condition (limb identity)	Internal validity — bias
20	Appropriate statistical tests used	Internal validity — bias
21	Valid and reliable measurement tools used (motion capture, force plates)	Internal validity-confounding (selection bias)
22	Appropriate adjustment for confounding variables	Internal validity-confounding (selection bias)
25	Loss of participants explained (if applicable)	Internal validity-confounding (selection bias)
27	Study has sufficient power (based on reported or implied sample size calculations)	Power

(Original Downs and Black Checklist is 27 items; items irrelevant to biomechanics intervention-free designs were removed or modified).

Table S7. Operational Definitions for Each Item

**Item No.**	**Operational definition used in this review**
1	Study states explicit aims related to ACLR gait asymmetry.
2	Kinematic or kinetic variables are clearly defined (e.g., peak knee moment).
3	Participant demographics, surgery details, and rehabilitation status are reported.
4	Walking task, speed, marker set, and force-plate setup described.
5	Study identifies key biomechanical confounders: prescribed speed, overground/treadmill, graft type, concomitant injuries, and time since surgery.
6	Effect estimates for ACLR vs. contralateral limb are provided.
7	Standard deviations, standard errors, confidence intervals reported.
10	Exact p-values or clear significance thresholds reported.
11	Sampling frame consistent with typical ACLR clinical cohorts.
12	Recruitment strategy clearly described and not selective.
15	Study explicitly states prospective data collection OR uses prospective registry.
16	Participants unaware of measurement purpose (rare in gait studies; scored conservatively).
18	Assessors blinded to limb identity during data processing (e.g., marker labeling anonymized).
20	Statistical analysis appropriate to design (e.g., paired t-tests, repeated measures).
21	Use of validated, calibrated 3D motion capture systems and force plates.
22	Adjusts for or controls key confounders (e.g., fixed speed trials).

25	All participants accounted for at analysis; missing data explained.
27	Provides sample size calculation OR sample size sufficient for biomechanical analysis.

Rationale for Using the Modified Downs and Black Checklist

The modified Downs and Black Checklist was selected because:

1. **It is suitable for non-randomized biomechanical studies**

   Most included studies were cross-sectional or observational within-participant comparisons of ACLR versus contralateral limbs. The original Downs and Black tool, widely used in physiotherapy, sports medicine, and biomechanics systematic reviews, is designed to assess reporting quality, external validity, internal validity (bias and confounding), and statistical power without assuming an interventional design.

2. **Modified Downs and Black tool allows domain-level mapping**

   Items in the Downs and Black Quality Index tool included domains such as confounding, selection, measurement of outcomes, and reporting. This allows transparent reporting while preserving methodological appropriateness for biomechanical studies.

3. **Consistency with prior gait-biomechanics systematic reviews**

   Recent biomechanical systematic reviews have used the Downs and Black tool or modified variants, making it a field-standard tool for ACL gait research.

Supplementary Material File S3: ***Summary of studies and time windows per outcome***

Table S8. Number of studies contributing to each outcome and each postoperative time window

Outcome	Total Studies (Overall)	Less than 12 months		Over 12 months	Meets Pooling Criterion?
		Short-Term (<6 mo)	Mid-Term (6–<12 mo)	Long-Term (≥12 mo)	
Peak knee flexion angle	19	4	6	9	Yes
Peak hip flexion angle	5	1	2	2	Yes
Peak vertical GRF	6	1	–	5	Yes
Peak knee flexion moment	11	1	2	8	Yes
Peak knee extension moment	13	1	6	6	Yes
Peak knee adduction moment	14	1	3	10	Yes

Supplementary Material File S4: Funnel plots indicating potential publication bias

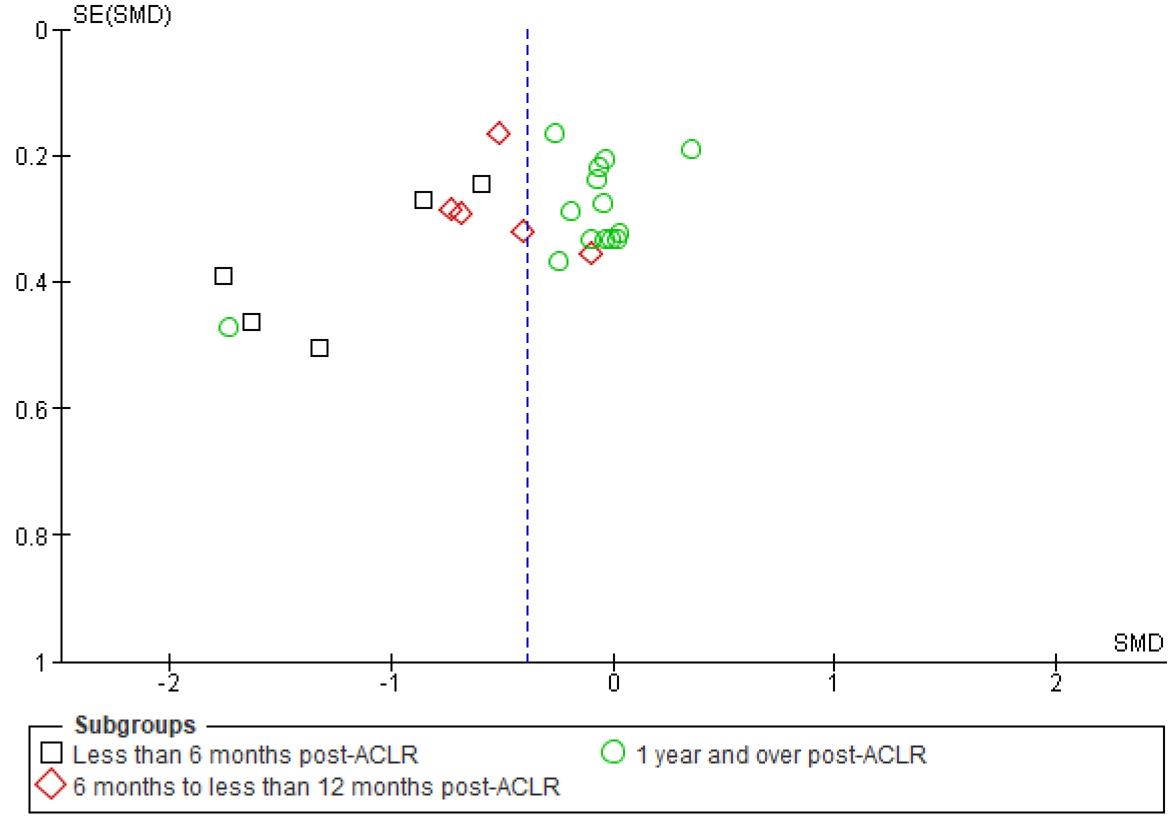

Figure S1. Funnel plot indicating potential publication bias for the parameter peak knee adduction angle.

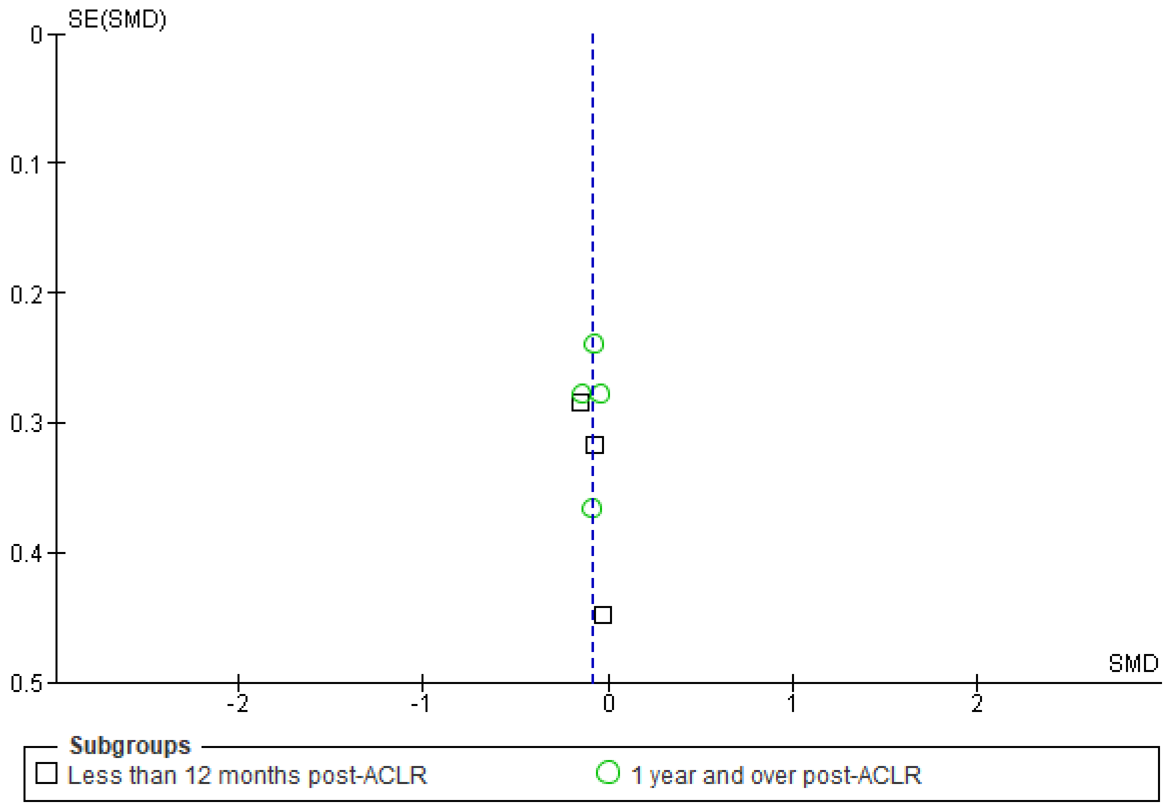

Figure S2. Funnel plot indicating potential publication bias for the parameter peak hip flexion angle.

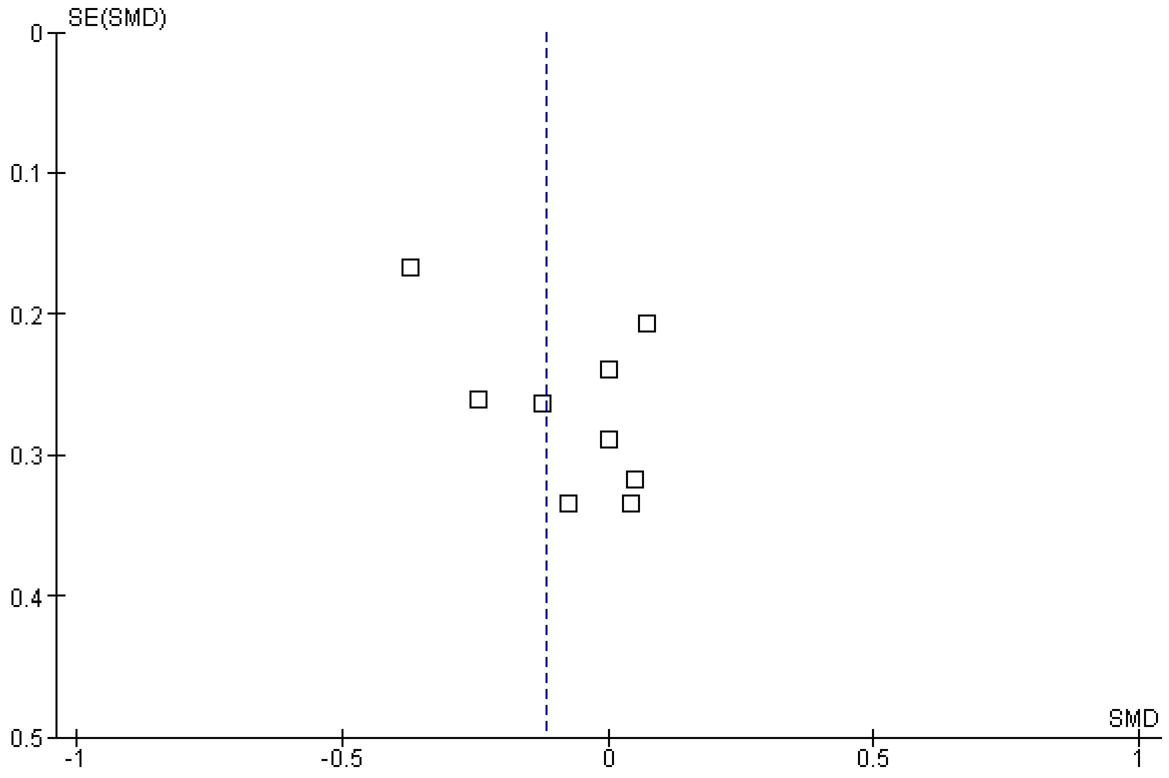

Figure S3. Funnel plot indicating potential publication bias for the parameter peak vertical ground reaction force.

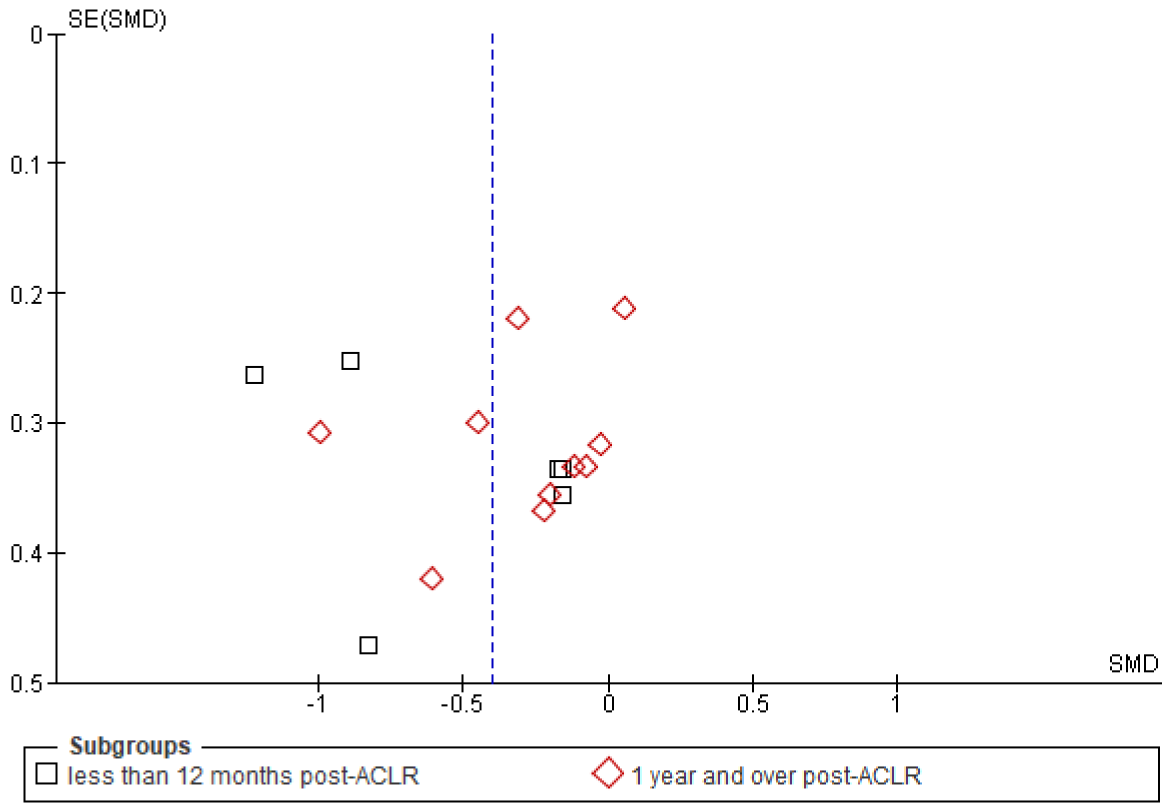

Figure S4. Funnel plot indicating potential publication bias for the parameter peak knee flexion moment.

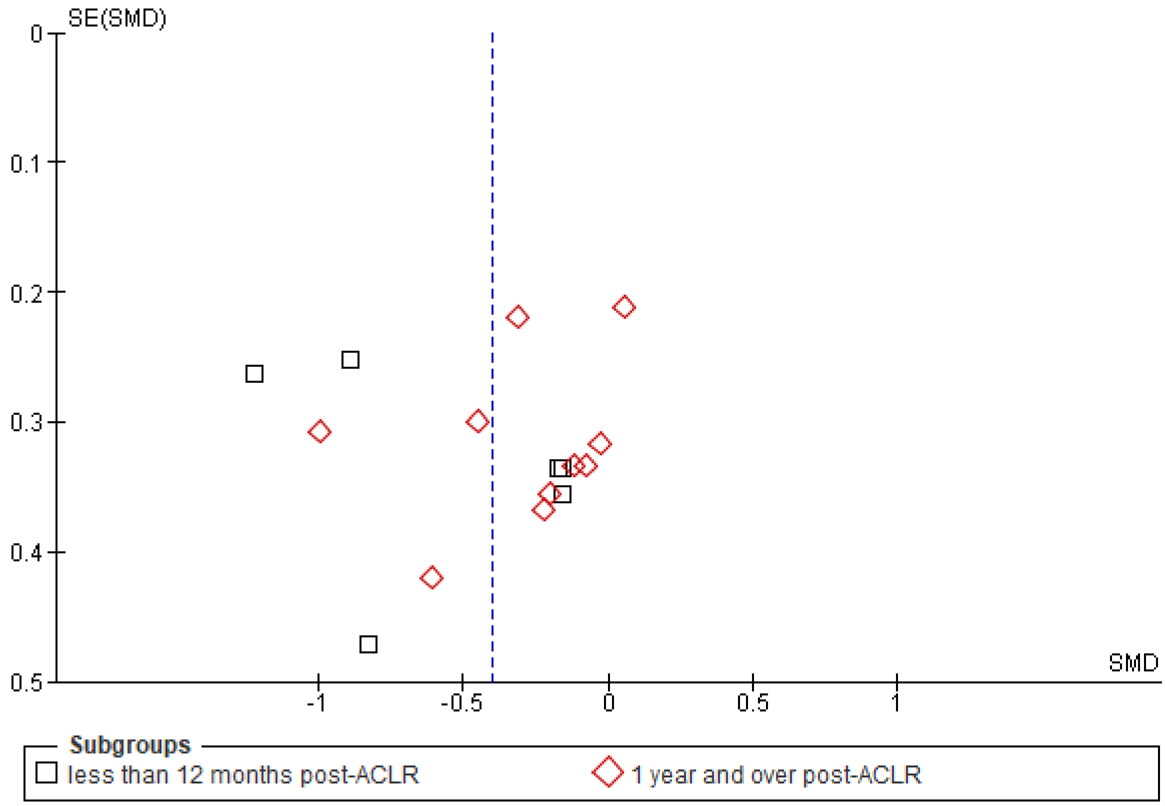

Figure S5. Funnel plot indicating potential publication bias for the parameter peak knee extension moment.

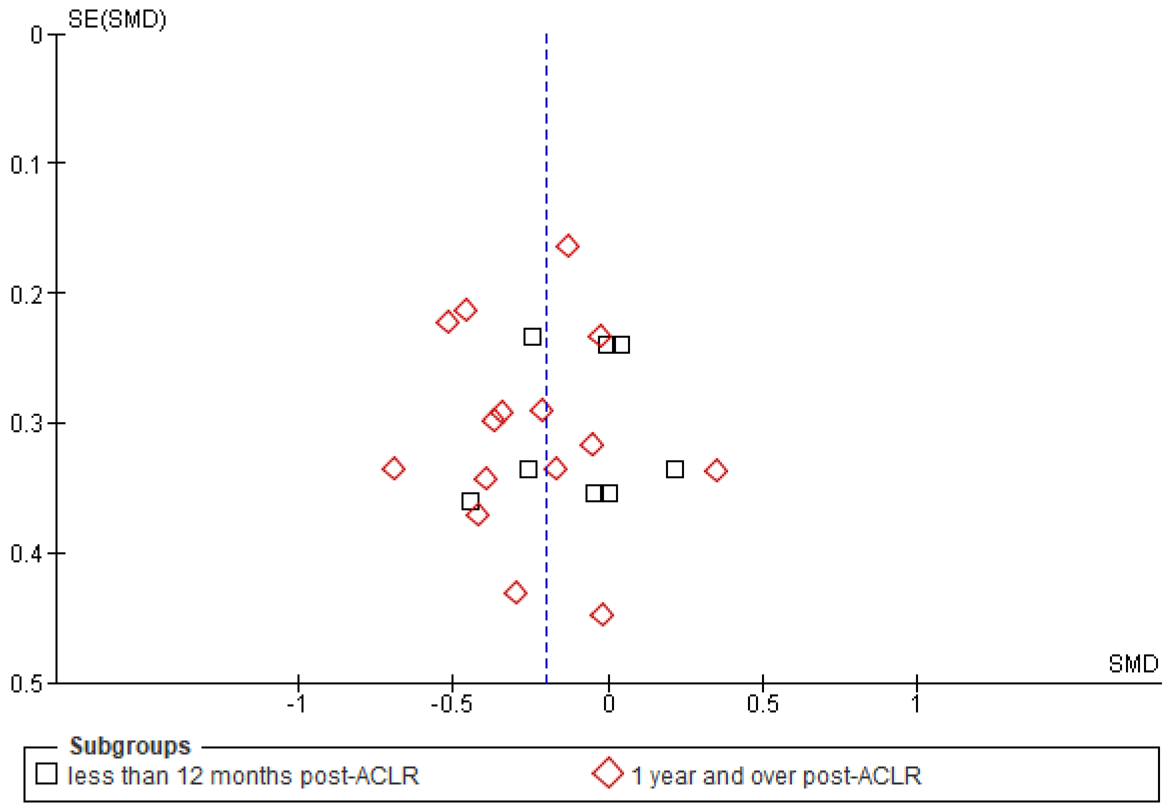

Figure S6. Funnel plot indicating potential publication bias for the parameter peak knee adduction moment.
